# Defect-Healed Carbon Nanomembranes for Enhanced Salt Separation: Scalable Synthesis and Performance

**DOI:** 10.1021/acsami.4c00252

**Published:** 2024-04-19

**Authors:** Zhen Yao, Pengfei Li, Kuo Chen, Yang Yang, André Beyer, Michael Westphal, Qingshan Jason Niu, Armin Gölzhäuser

**Affiliations:** † Physics of Supramolecular Systems and Surfaces, Bielefeld University, Bielefeld 33615, Germany; ‡ College of Chemical Engineering, China University of Petroleum (East China), Qingdao 266580, PR China; § Institute for Advanced Study, Shenzhen University, Shenzhen 518060, PR China

**Keywords:** carbon nanomembranes, size-selective defect sealing, interfacial polymerization, scalable 2D materials, desalination

## Abstract

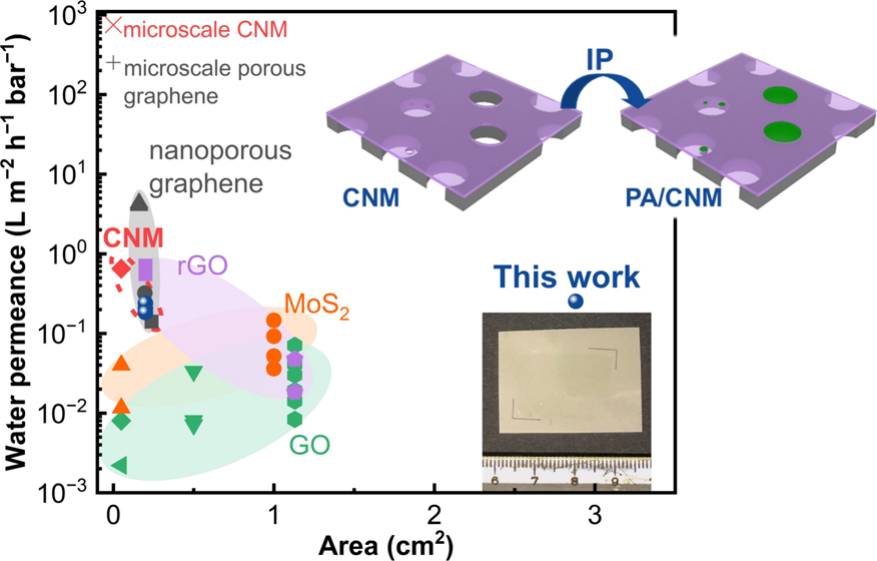

Carbon nanomembranes (CNMs), with a high density of subnanometer channels, enable superior salt separation performance compared to conventional membranes. However, defects that occur during the synthesis and transfer processes impede their technical realization on a macroscopic scale. Here, we introduce a practical and scalable interfacial polymerization method to effectively heal defects while preserving the subnanometer pores within CNMs. The defect-healed freestanding CNMs show an exceptional performance in forward osmosis (FO), achieving a water flux of 105 L m^–2^ h^–1^ and a specific reverse salt flux of 0.1 g L^–1^ when measured with 1 M NaCl as draw solution. This water flux is 10 times higher than that of commercially available FO membranes, and the reverse salt flux is 70% lower. Through successful implementation of the defect-healing method and support optimization, we demonstrate the synthesis of fully functional, centimeter-scale CNM-based composite membranes showing high water permeance and a high salt rejection. Our defect-healing method presents a promising pathway to overcome limitations in CNM synthesis, advancing their potential for practical salt separation applications.

## Introduction

Membrane separation based on two-dimensional (2D) materials has received increased interest in the past decade, especially in the fields of desalination and water treatment.^1^ 2D materials, *e.g.*, graphene,^2,3^ graphene oxide,^4,5^ carbon nanomembranes (CNMs),^6−9^ organic frameworks,^10^*etc*, with unique microstructures and properties allow an enhanced separation efficiency and structural stability compared to conventional membranes. Notably, ultrathin CNMs, generated from electron-induced cross-linking of a self-assembled monolayer (SAM) of terphenylenethiol (TPT) molecules on a gold surface, show an extremely high water permeability^6^ of 730 L m^–2^ h^–1^ bar^–1^ and an extraordinary salt selectivity of 0.09 g L^–1^ in FO.^7^ These favorable properties stem from a dense network of nanochannels (pore diameter of 0.7 nm) with an excessively high density of 10^18^ m^–2^.^6^ In fact, the water permeability per channel in CNMs (∼66 water molecules s^–1^ Pa^–1^)^6^ is comparable to that of aquaporins and carbon nanotubes,^11−13^ rendering CNMs highly attractive for water treatment applications. However, as with other 2D materials,^2,3,14,15^ the occurrence of defects, such as nonselective pores and larger-scale tearing during preparation and transfer have limited their large-scale production.

There have been various efforts in mitigating and sealing defects in 2D membranes, *e.g.*, stacking multiple layers of continuous membranes,^7,15^ atomic layer deposition,^14^ filtering a suspension of particles,^16^ selective electrochemical deposition,^17^*etc.* These methods, although facile and convenient, present problems of water flux loss and possible instability of the sealing material. Another strategy involves defect healing with permeable materials formed by novel interfacial polymerization (IP). The group of R. Karnik reported large-scale tear healing of a graphene membrane by the selective interfacial polymerization of Nylon-6,6.^14,18−21^ Hence, a selective interfacial polymerization at defects would allow a scalable preparation of nanoporous 2D membranes.^14,22^ Yet, up to now, only IP at porous graphene grown by chemical vapor deposition has been studied. The pores in graphene are created by a combination of physical and chemical treatments,^2,23^ and the following IP method involves a polymer formation inside the pores of a support layer beneath the defect.^14,18−22,24^ With polymers plugging inside the support pores, the top separation layer fails to form a continuous film, and the interface between graphene and the support layer will be at risks of delamination and leakage in high-pressure driven applications.

To overcome these challenges, here we present a practical and scalable method to prepare centimeter-sized nanoporous CNMs using selective interfacial polymerization of m-phenylenediamine (MPD) and trimesoyl chloride (TMC) to heal defects of areas from the nanometer to the centimeter scale. We demonstrate the application of these defect-healed CNMs in FO by using NaCl as the draw solution (DS). Through the successful implementation of our defect-healing method, we achieve the scalable synthesis of centimeter-scale CNM-based composite membranes, showing the potential of CNM-based membranes for practical water treatment applications.

## Results and Discussions

**Figure 1:**
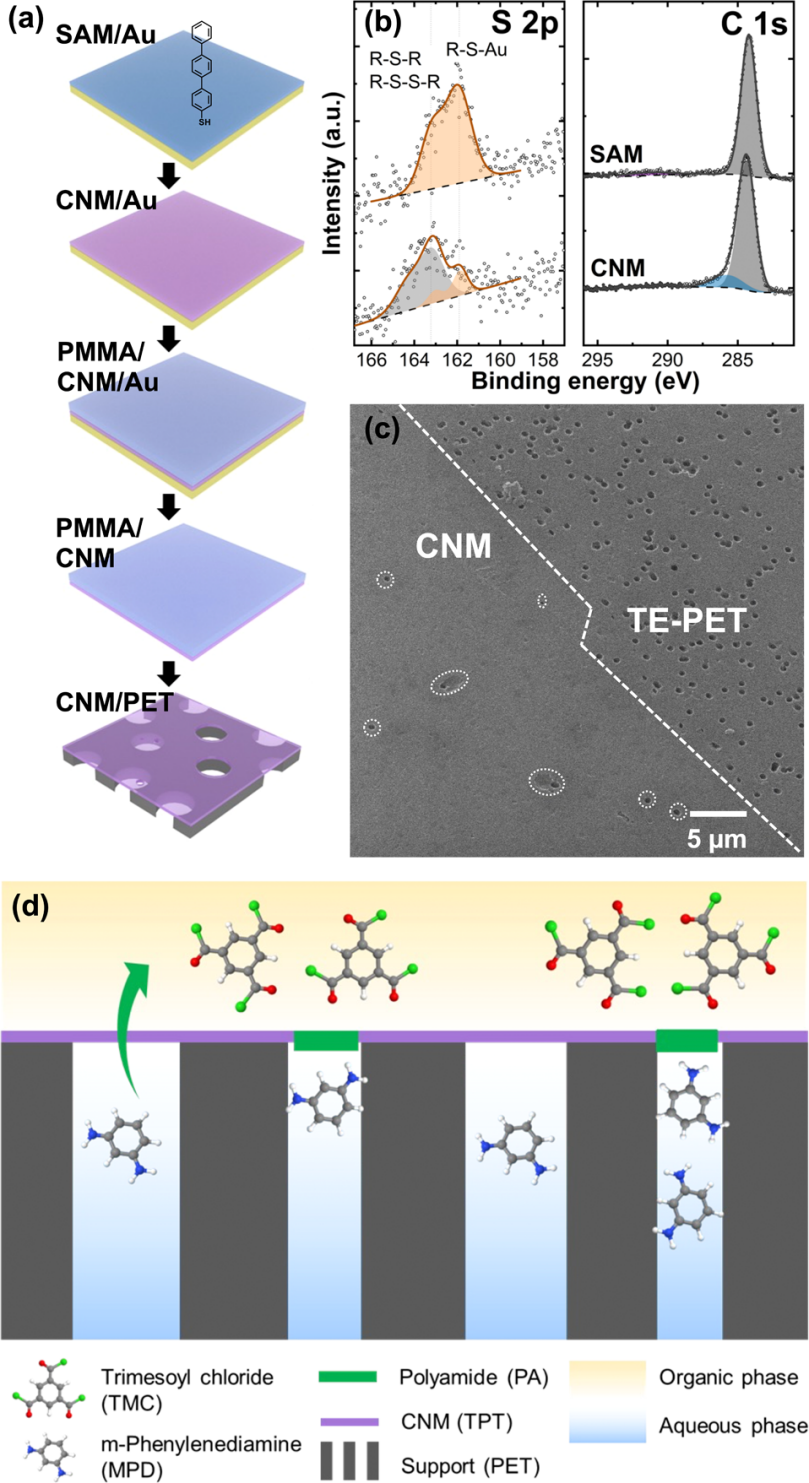
CNM fabrication and defect-healing procedure. (a) TPT-CNM, formed from electron-induced cross-linking of TPT-SAM/Au(111), is transferred to a TE-PET support *via* a PMMA-assisted transfer procedure. (b) S 2p and C 1s XPS spectra of TPT-SAM/Au and TPT-CNM/Au. (c) HIM image of TPT-CNM on TE-PET with the dashed line showing the edge and dotted lines highlighting the micrometer-scale defects. (d) Schematics of polyamide formation at the defects from MPD in the aqueous phase and TMC in the organic phase.

**Figure 2:**
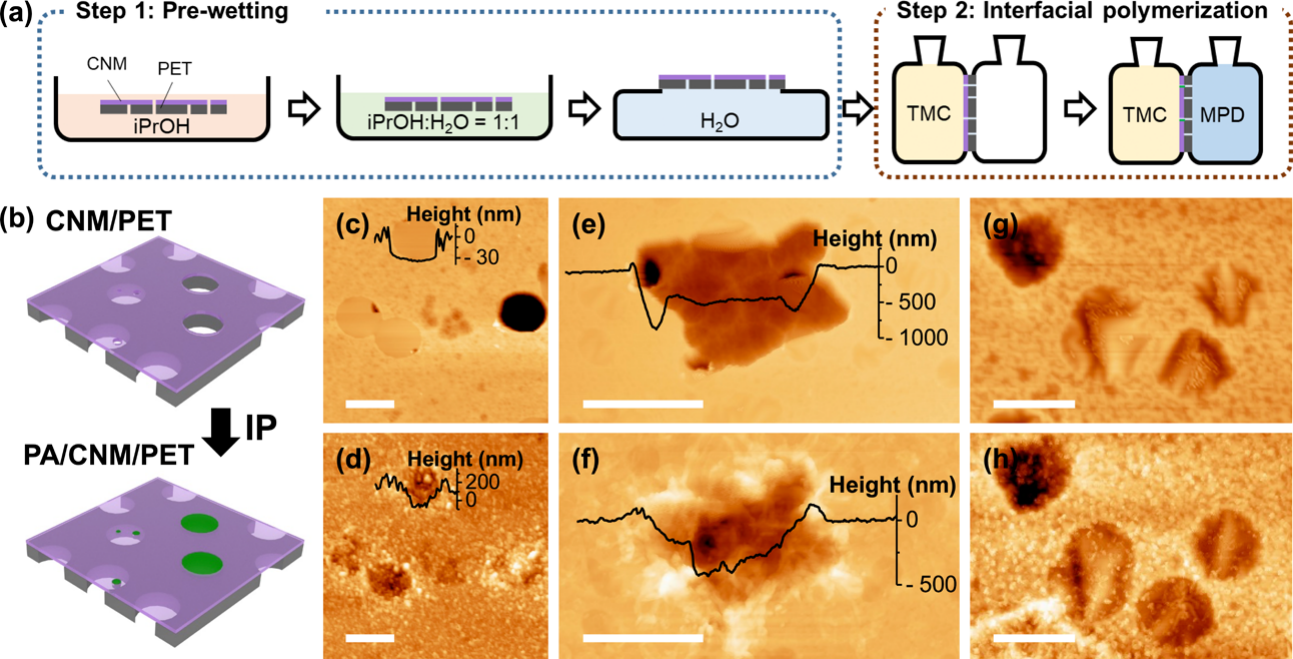
Interfacial polymerization to heal defects in the CNM. (a) Schematics showing the IP steps. The CNM/PET composite was first prewetted *via* a sequential exposure to isopropanol, isopropanol/water 1:1 mixture, and water. The wetted membrane was then mounted in a home-built cell for IP. The TMC/hexane was introduced on the CNM side of the composite. When no leakage was observed, MDP/water was added to the PET side allowing polyamide formation at the defects. (b) Schematics of the PA formation precisely at defects without blocking nanopores (<0.5 nm) in CNM. (c–h) Topographic AFM images of TPT-CNM transferred onto TE-PET (c,e,g) and the same location after IP of MPD and TMC (d,f,h) (scale bar = 3 μm). The insets in (c–f) show the height profiles.

For our interfacial polymerization experiments, we choose CNMs made by electron-induced cross-linking of SAMs of TPT molecules on a Au(111) surface that in earlier work demonstrated high water permeability^6,25,26^ and selectivity.^7^ First, TPT molecules adsorb onto the gold substrate *via* the thiol group to form an ordered and densely packed SAM,^27,28^ as illustrated by the S 2p and C 1s signals from X-ray photoelectron spectroscopy (XPS) (Figure 1a,b). The subsequent electron irradiation cleaves C–H and Au–S bonds so that the molecules get cross-linked with their neighboring molecules to form a continuous film, *i.e.*, the CNM.^29^ The cross-linking is confirmed by the appearance of an organosulfide (R–S–R and R–S–S–R) peak at 163.3 eV (Figure 1b). The C 1s intensity indicates that the carbon content is reduced by 2% after cross-linking, which corresponds to a membrane thickness of approximately 1.2 nm.^30^ The CNM was then transferred onto a track etched polyester (TE-PET) support *via* a polymer-assisted transfer procedure^7,30−34^ to minimize damages to the membrane (Figure 1a). The TE-PET supports have well-defined isolated pores,^35^ which will allow effective decoupling of the CNM property from the support behavior. Successful transfer was confirmed using both helium ion microscopy (HIM) and atomic force microscopy (AFM), as shown in Figures 1c and 2c,e,g. The TE-PET pores covered with the CNM appear bright in the HIM image, while noncovered pores appear as dark spots, highlighted by dotted lines (Figure 1c). The CNM-covered pores are more discernible in AFM. As shown in Figure 2c,e, a CNM suspends over the pores with a subsidence of 30–50 nm, whereas noncovered pores usually show a depression of several hundred nanometers. It is noted that such subsidence behavior is typical for ultrathin membranes transferred onto substrates with open pores due to van der Waals interactions between the pore’s walls and the membrane.^9,36^

Defects in CNMs represent nonselective pores, varying in size from nanometers to micrometers. The nanoscale defects are mainly formed during the CNM preparation process and hence are classified as intrinsic defects. When examining the transferred membrane, characterized by its ultrathin nature and low mechanical stability, we observe partially covered or completely open TE-PET pores (Figure 1c). In addition to the intrinsic defects, the CNM/PET composites also often exhibit cracks and delamination caused by handling and transfer (Figure S1), along with large tears emerging at etch pits on the PET surface (Figures 1c and 2e). The etch pits, featuring hundreds of nanometers of corrugations on the PET surface, are inherent to the PET fabrication process.^37,38^ Despite constituting less than 1% of the total area, CNMs in these regions typically exhibit defects.

To heal those defects or tears in the transferred CNM, we developed a scheme using interfacial polymerization with MPD and TMC (Figure 1d). Since the solubility of MPD in hexane is higher than that of TMC in water, the amine monomers stored in the TE-PET pores first diffuse through the defects to the hexane phase to polymerize with TMC. When MPD monomers further diffuse to the oil phase, a nodular primary layer is formed. The following increase in cross-linkage and thickness of the polyamide (PA) layer limits further diffusion of the MPD monomers, which then leads to the termination of the polymerization reaction. Given the size of MPD (0.5–0.6 nm), it is hypothesized that pores or defects larger than 0.6 nm will be completely sealed by PA while those nanochannels in the CNM with a size smaller than 0.5 nm will remain open. According to the IP mechanism, the actual polymerization interface is pinned to the hexane phase. Therefore, in order to precisely control the position of the IP without damaging the CNM, we use a two-step procedure (Figure 2a). First, we fill the TE-PET channels with water *via* a sequential exposure to isopropanol, an isopropanol–water mixture, and water. After this prewetting step, the TMC/hexane solution is introduced to the CNM side of the composite membrane. The wetting of the hexane in the TE-PET pores in this case is stopped by the prefilled water inside the channel. When no hexane leakage is observed, the MPD/water solution is added on the PET side to allow interfacial polymerization. Using this method, we managed to fix the water/hexane interface on the CNM side of the composite. Figure 2c–h shows AFM images of the PA formation precisely at the defects without interfering with the integrity of the CNM. The polymer can form at both completely and partially open TE-PET pores (Figure 2c,d) and also at pores inside etch pits on PET (Figure 2e,f). Figure 2g,h illustrates polymers appearing within the freestanding CNM, suggesting the healing of nanometer sized (>0.6 nm) pores in the CNM. The topographic evaluation of the PA/CNM/PET composites confirms that interfacial polymerization is capable of repairing defects in CNM/PET composites ranging from 0.6 nm to several micrometers.

**Figure 3:**
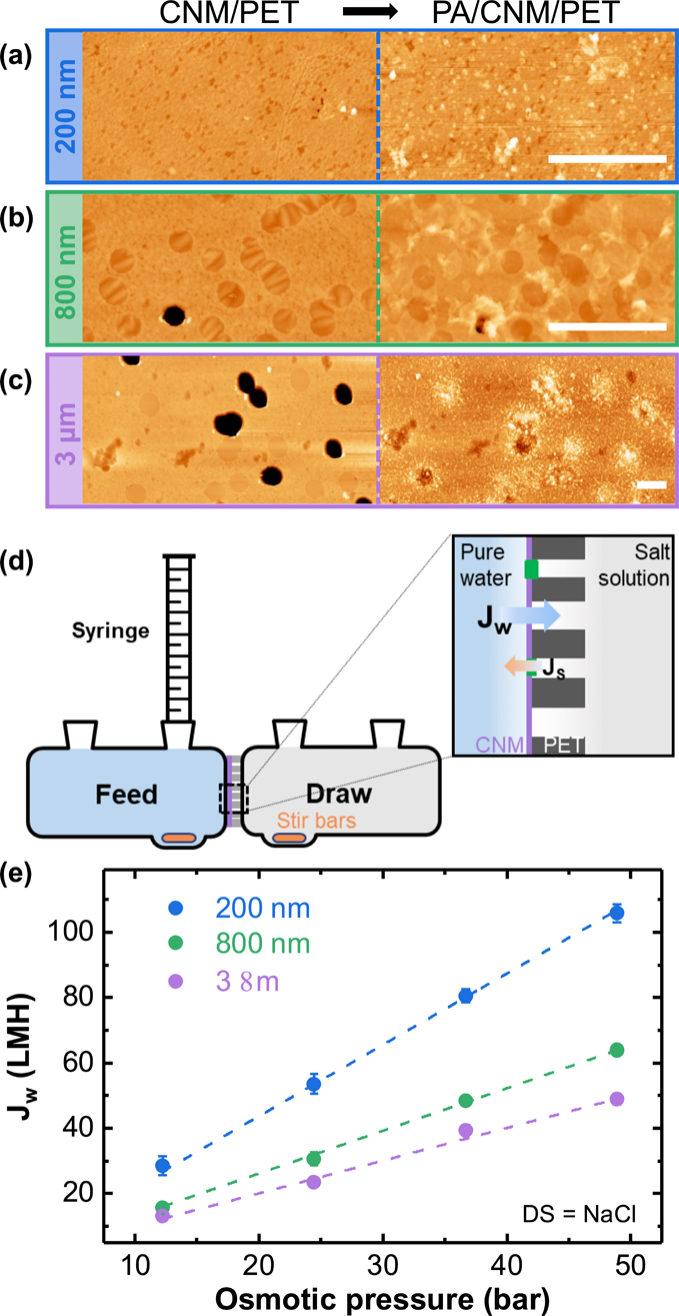
Forward osmosis experiments with PA/CNM/PET. (a–c) AFM images of the CNM on TE-PET supports with different pore sizes before and after IP (scale bar = 4 μm). (d) Schematics of the permeation cell with the inset showing an effective osmosis process with PA/CNM/PET composites. (e) Water flux (*J*_w_) across the freestanding areas in the PA/CNM/PET composites as a function of osmotic pressures (calculated from the salt solution concentrations using the Van’t Hoff equation). Each data point represents an average of at least three independently prepared samples, with the error bar showing the standard deviation. The dotted lines represent linear fit curves for PA/CNM/PET composites with different PET pore sizes. The fitting parameters are listed in Table 1.

Next, we examined the separation performance of the PA/CNM/PET composites in the FO operation. An FO separation test was designed using TPT-CNMs transferred onto the TE-PET support with well-defined and isolated pores of diameters ranging from 200 nm to 3 μm (Figure 3a–c). Similar to nanoporous graphene membranes,^39^ the fraction of defects increases with PET pore size (see also S1 for details) and is 0.8%, 14%, and 52% for CNM on TE-PET with pores of 200 nm, 800 nm, and 3 μm diameter, respectively, indicating a higher probability of the CNM to rupture when it is suspended over larger pores. After IP, no open PET pores are visible, indicating successful defect healing (Figure 3a–c). Note that the CNM morphology can still be seen alongside the polymer structure, especially in the case of TE-PET with 200 nm pores (Figure 3a), confirming the reliability and reproducibility of the defect healing process.

The FO measurements with PA/CNM/PET composites were carried out in a customized glass cell with two compartments, as schematically illustrated in Figure 3d. The composite membrane with an effective area of ∼0.2 cm^2^ was fixed in the cell between a feed solution (FS) of deionized (DI) water and a DS of NaCl with concentrations of 0.25–1 M. NaCl was selected due to the size of hydrated Na^+^ and Cl^–^ (0.716 and 0.664 nm)^40^ that match the AFM determined mean pore size of 0.7 nm in TPT-CNM.^6^ Therefore, owing to size exclusion,^7^ TPT-CNMs show a rejection of NaCl, while water molecules (∼0.28 nm)^41^ can still pass.

For an effective FO, a high water flow from the feed side to the draw side and a low reverse salt flux from the draw side to the feed side (Figure 3d) are needed. We evaluated the FO performance of the composite membranes by measuring the volume of water that flows to the draw side and the conductivity of the feed solution after 20 min. The feed solution conductivity quantifies the amount of salts that pass through the membrane. Further details of the measurements are provided in S2.1.

In CNM/PET composites without IP, there are many micrometer scaled defects present in the membrane (Figures 1c and 3a–c), and therefore, we did not observe any FO water flow. Following the healing of defects through IP, we tested the composite with NaCl solutions of varying concentrations to generate different osmotic pressures. To ensure that the CNM and polymers were not damaged during the FO, we examined the composite with HIM after the measurements (Figure S5).

As shown in Figure 3e, the water flux in the PA/CNM/PET composites increases linearly with the osmotic pressure (*i.e*., salt concentration). The extracted water permeance (*P*_w_) together with water flux (*J*_w_) and specific reverse salt flux (*i.e.*, salt flux/water flux *J*_s_/*J*_w_) for 1 M NaCl DS are listed in Table 1. The composite with 200 nm PET pores shows the highest water flux with a water permeance of 2.2 L/m^2^/h/bar (LMH bar^–1^) for freestanding PA/CNM. It is noted here that the orientation of the membrane, *i.e.*, CNM-facing-the-feed-solution or CNM-facing-the-draw-solution, has an influence on the water flux due to the internal concentration polarization occurring inside the PET support (Figure S4).^42,43^ For simplicity, we present and discuss exclusively the values obtained with the CNM-facing-the-feed-solution configuration throughout the remainder of this paper. With 1 M NaCl as DS, the water flux reaches 105 LMH, ten times higher than that for commercially available cellulose triacetate (CTA) membranes,^44−47^ and the specific reverse salt flux is 0.1 g L^–1^. These values agree with the water permeance of ∼13 LMH bar^–1^ and a selectivity of 0.09 g L^–1^ reported previously for a bilayer CNM on TE-PET.^7^ As the PET pores get larger, the fraction of defects on CNM/PET increases (Figure S1), leading to more freestanding parts being clogged by PA. Since PA allows lower water flux and higher reverse salt flux than the CNM (see S3 for details), the PA/CNM/PET composite with larger PET pore size yields a lower water flux and salt selectivity. For the composite with 800 nm and 3 μm PET pores, when measured with 1 M NaCl as the DS, the water flux is 64 and 49 LMH for freestanding PA/CNM, respectively, and the salt selectivity is 0.11 and 0.3 g L^–1^, respectively.

**Table 1:** Forward Osmosis Performance for Freestanding PA/CNMs Evaluated on PA/CNM/PET Composites with Different PET Pore Sizes

PET pore size/μm	areal porosity^a^/%	defect fraction^a^/%	P_w_^b^/LMH bar^–1^	DS = 1 M NaCl	
				*J*_w_^b^/LMH	*J*_s_/*J*_w_/g L^–1^
0.2	9 ± 4	0.8 ± 0.4	2.2	105 ± 3	0.10 ± 0.06
0.8	18 ± 3	14 ± 5	1.3	64 ± 1.5	0.11 ± 0.01
3	18 ± 1	52 ± 24	1.0	49 ± 2	0.34 ± 0.16

a The areal porosity and defect fraction are evaluated from at least nine AFM topographic images (recorded from three different locations at three independently prepared samples). The errors represent standard deviation. See S1 for details about the evaluation.

b *P*_w_ and *J*_w_ are values of freestanding membranes.

**Figure 4:**
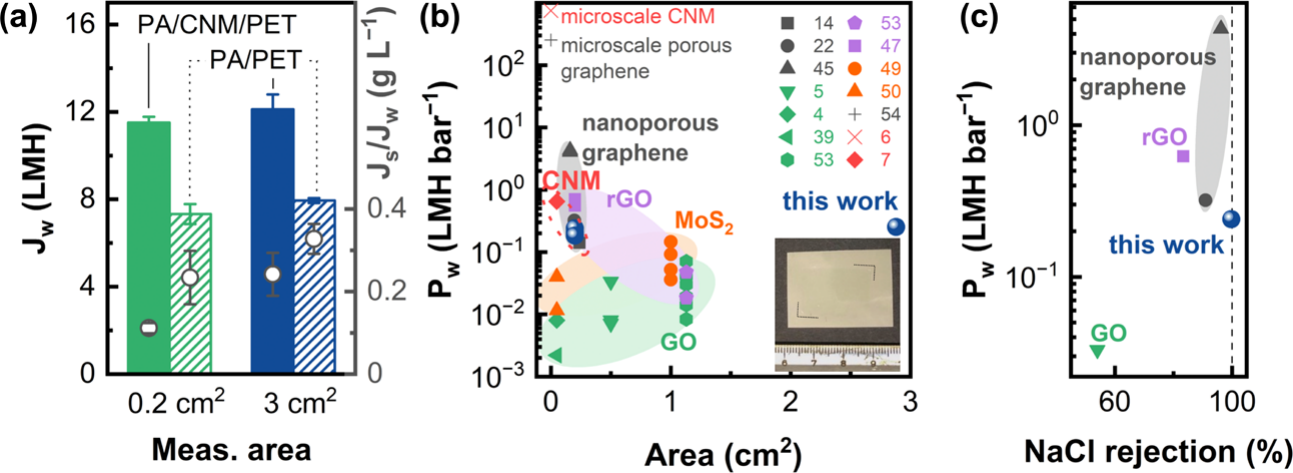
Upscale production of PA/CNM/PET. (a) Comparison of water flux (*J*_w_) and specific reverse salt flux (*J*_s_/*J*_w_) for PA/CNM/PET and PA/PET composites with measured areas of 0.2 and 3 cm^2^. Each data point represents the average value of three independently prepared samples with the error bar showing the standard deviation. Comparisons of (b) water permeance and (c) salt rejection measured during FO for PA/CNM/PET composites in this work with other 2D material-based membranes in the literature. The inset in (b) shows a 3 cm^2^ PA/CNM/PET composite. Note that we only compare the salt rejection measured *via* FO with a DS of nonsalt and a FS of NaCl in (c). The salt rejection values for the membranes measured *via* reverse osmosis are listed in Table S1.

Here, we note that freestanding PA/CNM allows a significantly higher water flux (∼100 LMH, DS = 1 M NaCl) compared to state-of-the-art flatsheet thin-film composite (TFC) FO membranes, which typically demonstrate a water flux of 10–40 LMH and a salt selectivity of 0.2–0.6 g L^–1^ with 1 M NaCl as the DS.^46,48^ However, as with other 2D materials,^4,5,7,14,22,39,45,49,50^ the membrane is fragile on the macroscopic scale and cannot be handled without a supporting layer. Depending on the support used, usually, the effective membrane area represents less than 20% of the total area. From a practical point of view, it is essential that these 2D material-based membranes not only demonstrate superior material performance compared to conventional ones, but also undergo comprehensive evaluation, treating support and 2D materials as an integrated system conducive to scalable production.^1,51^ This requires a careful selection of the support material to maximize the area of the freestanding membrane while preserving their integrity.^39^ Therefore, from the three TE-PET supports used in this study, we chose PET with 800 nm pores that possesses the highest freestanding CNM areal percentage of ∼15% for the scalability test (Figure S1e). In our small glass cell test with an exposed membrane area of 0.2 cm^2^, this composite exhibited an overall water flux of 11.5 LMH (DS = 1 M NaCl) with a specific reverse salt flux of 0.11 g L^–1^. Scaling up the composite from 0.2 cm^2^ to approximately 3 cm^2^ yielded a consistent water flux for both PA/CNM/PET and PA/PET composites, as illustrated in Figure 4a, indicating the scalability and reproducibility of our defect-healing method. Only a marginal increase in the reverse salt flux was observed. The specific reverse salt flux increased from 0.11 g L^–1^ to 0.24 g L^–1^ for the scaled-up membrane, possibly attributed to localized incomplete healing in the larger composite. Further details on the performance evaluation of the large composite are provided in S2.2.

Figure 4b,c provides a detailed comparison of water permeance and salt rejection for PA/CNM/PET composites with other 2D material-based membranes as reported in the literature. Obtaining large-areas of 2D materials is technically challenging owing to the limitations in synthesis scale, transfer, and defect mitigation.^1^ Therefore, most 2D material-based membranes reported have working areas on the millimeter to centimeter scale (Figure 4b). Among various membranes, the CNM, characterized by its nanoporous nature intrinsic to the electron-induced cross-linking process, demonstrates a higher water permeance than most other 2D laminate membranes, *i.e.*, graphene oxides (GOs),^4,5,52,53^ reduced graphene oxides (rGOs),^47,53^ and functionalized MoS_2_^49,50^ (Figure 4b). In terms of material properties, the high nanopore density of CNMs (∼10^18^ m^–2^)^6^ allows a comparable water permeance as nanoporous atomically thin graphene membranes^54^ on the micrometer scale. As a consequence, our PA/CNM/PET composites show a water flux comparable to those nanoporous graphene (NG) composites.^14,45,55^ Owing to the more uniform pore size distribution in the CNM than that in NG, our composite shows a NaCl rejection of 99.8%, which is higher than all other NG-, GO-, and rGO-based composites (Figure 4c) and comparable to the commercially available CTA membrane^45^ and state-of-the-art TFC membranes.^44,46,48^ Interestingly, our PA/CNM/PET composite with an overall water flux of 12 LMH under FO using 1 M NaCl against DI water lies also in the flux range for TFC FO membranes.^46,48^

## Conclusions

In summary, we have developed a simple and scalable approach to fabricating large-area defect-free nanoporous membranes for salt separation. Defects in centimeter-sized CNM/PET composites were successfully repaired by interfacial polymerization of polyamide using MPD as the aqueous phase monomer and TMC as the oil phase monomer. By optimizing the pore size and areal porosity in supports of track-etched polyester, defect-healed CNMs exhibit a specific reverse salt flux as low as 0.1 g L^–1^ coupled with a water flux of up to 105 LMH in forward osmosis with 1 M NaCl as draw solution. The successful upscaling of the defect-healed CNM composites to ∼15 times the original area demonstrates the scalability and reproducibility of our defect healing method. These functional centimeter-scale CNM-based composite membranes not only match the water permeance of commercially available FO membranes but also show an outstanding salt rejection of 99.8%. Our work, therefore, offers a facile and scalable route toward the mass production and practical applications of nanoporous membranes. This advancement, combined with our prior efforts in scalable CNM synthesis,^56^ will enable the development of more efficient and stable CNM-based water treatment technologies.

## Materials and Methods

### Carbon Nanomembrane Preparation

CNMs were prepared from electron-induced cross-linking of a self-assembled monolayer of 1, 1′, 4′, 1′-terphenyl-4-thiol (TPT, Sigma-Aldrich) on Au/mica substrates (Georg Albert PVD Deposition, Germany). The detailed SAM preparation procedure is described elsewhere.^30^ After self-assembly, TPT-SAMs were converted into TPT-CNMs with 100 eV electrons at a dose of 50 mC cm^–2^ in a high vacuum chamber (<8 × 10^–8^ mbar). The CNM prepared was stored under argon until further use.

### Transfer

CNM transfer to track etched polyester supports (ipPORE, hydrophobic, standard thickness 20–23 μm, it4ip S.A., Belgium) was performed *via* a polymer-assisted transfer procedure. The full details can be found in our previous studies.^7,30−33^ Here, briefly, we coated the CNM with a layer of poly(methyl methacrylate) (PMMA, AR-P 671.04) in ethyl acetate (Fisher Chemical) to stabilize the CNM during the transfer process. The PMMA/CNM/Au stack was delaminated from the mica support by dipping into water and the Au film was fully etched by floating the stack on I_2_/KI/H_2_O (1:4:10; iodine, 99.8%, Alfa Aesar; potassium iodide, ≥99%, Carl Roth) solution. Next, the PMMA/CNM stack was transferred onto a PET support followed by >10 h 120 °C baking in air to fully remove the water molecules at the CNM/PET interface. The PMMA-coating was dissolved *via* 1h immersion in acetone (≥98%, Fisher Chemical). The heating step ensures a good adhesion between the CNM and PET support. No delamination was observed during PMMA dissolution, IP and FO test.

### Interfacial Polymerization

IP was performed in a home-built polycarbonate cell using 0.02 g/mL 1,3-phenylenediamine (MPD, >98%, TCI) aqueous solution and 0.0015 g/mL 1,3,5-benzenetricarbonyl trichloride (TMC, 98%, Sigma-Aldrich) in hexane (≥97%, VWR) for 1 min. 0.001 g/mL sodium dodecylbenzenesulfonate (SDBS, technical grade, Aldrich) was added into the MPD solution to regulate the amine transportation producing more homogeneous polyamide.^24^ To ensure that polymerization occurs only at the defects, we adopted a two-step procedure for IP as illustrated schematically in Figure 2a. The CNM/PET composite was prewetted *via* a sequential exposure to isopropanol (≥99.7%, VWR Chemicals), isopropanol/water (1:1) and deionized (DI) water. The wetted membrane was then mounted in the cell, with TMC/hexane introduced to the CNM side of the composite. When no leakage was observed, MDP/water was added to the PET side, allowing PA formation precisely at the defects. After IP, the membrane was heat-cured at 60 °C for 5 min to further increase the cross-linking degree of PA.

### Characterization

X-ray photoelectron spectra for TPT-SAMs/Au and TPT-CNM/Au were acquired using an Omicron multiprobe spectrometer with a monochromatic Al K_α_ source (1486.6 eV) and pass energy set to 25 eV. The spectra were fitted by using CasaXPS software with all binding energies referenced to the Au 4f_7/2_ peak (84.0 eV).^57^ Shirley and linear backgrounds were used for C 1s and S 2p spectra, respectively, and Gaussian/Lorentzian functions (GL30) were used for the curve fitting.

The helium ion microscope image of CNM/PET was acquired with a Carl Zeiss Orion Plus instrument with a He^+^ beam energy of 30 kV and a beam current of ∼0.2 pA. Atomic force microscope images of CNM/PET and PA/CN/PET composites were obtained using a NT-MDT instrument (Ntegra) in tapping mode with a Tap 150Al-G sensor (Budget Sensors, *k* ≈ 5 N m^–1^, *f*_0_ ≈ 150 kHz) under ambient conditions.

### Forward Osmosis Measurement

The FO measurements with a membrane area of 0.2 cm^2^ were performed with a 2 mL side-by-side glass cell (PermeGear, Inc., 5 mm orifice) using deionized water as the feed solution and sodium chloride (99.5–100.5%, VWR Chemicals) as the draw solution (schematics shown in Figure 3d). The details of the measurement are provided in S2. Briefly, the composite membrane was sandwiched between two glass cells, with the CNM side facing the feed solution. A 100 μL syringe (Hamilton 700) was attached to one port of the feed cell, and a Teflon plug was used to seal the other. During the measurement, the feed side was filled with DI water, while the draw side was filled with 0.25–1 M NaCl. The osmotic pressure drives the water transport from the feed side to the draw side, which leads to a drop in the water meniscus level in the syringe. The water flux *J*_*w*_ was then derived following *J*_w_ = *k*/(*A*·Ø), where *k* is the slope of the volume change against time, *A* is the area of the cell orifice, and Ø is the PET porosity (Ø = 1 when considering the composite performance).

The water permeance *P*_w_ was determined from *P*_w_= *J*_w_/Π, where the osmotic pressure Π from NaCl solution was calculated using the van’t Hoff equation, Π *= ic*R_g_*T* with a van’t Hoff factor *i* = 2 for NaCl, ideal gas constant R_g_*=* 0.082 L bar K^–1^ mol^–1^, and temperature *T* = 293.15 *K*.

The reverse salt flux *J*_s_ was calculated from the increase in the salt concentration of the FS as determined from the conductivity,
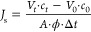
1where, *V* and *c* denote the volume and concentration of the FS, respectively; Δ*t* represents the measured time.

NaCl rejection was measured by a 24 h test using 25 wt % glycerol ethoxylate (average Mn ∼ 1000, Aldrich) as the DS and 20 mM NaCl as the FS. The rejection *R* was calculated from^14,22^
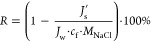
2where,  is the salt flux from the FS to the DS determined using eq 1 ; *J*_w_ is determined from the first hour of FO; *M*_NaCl_ is the molecular weight of NaCl; *c*_f_ is the mole concentration of the FS.

The FO measurements with a membrane area of 3 cm^2^ were performed with a home-built cross-flow FO system (details provided in S2). DI water was used as the feed solution and 1 M NaCl was used as the DS, circulating at a flow velocity of 0.1 m/s. Both CNM-facing-the-FS and CNM-facing-the-DS configuration was tested. The water flux *J*_w_ was determined by measuring the weight loss of the FS,
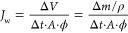
3where, Δ*V* and Δ*m* are the volume and mass changes of the feed solution, respectively; ρ is the FS density; Δ*t* is the measured time. The reverse salt flux *J*_s_ was calculated according to eq 1.

## Supporting Information Available

Defect fraction of CNM/PET (S1); forward osmosis measurements including setups for different membrane sizes and different membrane orientations (S2); comparisons of PA/CNM/PET and PA/PET (S3); comparisons to other 2D material-based membranes (S4) (PDF)

## Abbreviations

CNM: carbon nanomembrane
FO: forward osmosis
2D: two-dimensional
TPT: terphenylenethiol
IP: interfacial polymerization
MPD: m-phenylenediamine
TMC: trimesoyl chloride
XPS: X-ray photoelectron spectroscopy
TE-PET: track etched polyester
HIM: helium ion microscopy
AFM: atomic force microscopy
PA: polyamide
FS: feed solution
DI: deionized
DS: draw solution
TFC: thin-film composite
PMMA: poly(methyl methacrylate)
