# Supplementary material for: Defect-Healed Carbon Nanomembranes for Enhanced Salt Separation: Scalable Synthesis and Performance
Source: ACS Appl Mater Interfaces. 2024 Apr 19;16(17):22614–21. doi: 10.1021/acsami.4c00252 (PMC11073045; doi:10.1021/acsami.4c00252)
Supplement: Supplementary file 1 — am4c00252_si_001.pdf [file am4c00252_si_001.pdf]

Supporting Information to

Defect-Healed Carbon Nanomembranes for Enhanced Salt Separation: Scalable Synthesis and Performance

Zhen Yao,[†,**] *Pengfei Li,*[†,‡,**] *Kuo Chen,*[†,‡] *Yang Yang,*[†] *André Beyer,*[†] *Michael Westphal,*[†] *Qingshan Jason Niu,*[§,*] *and Armin Gölzhäuser*[†,*]

[†]Physics of Supramolecular Systems and Surfaces, Bielefeld University, 33615 Bielefeld, Germany
[‡]College of Chemical Engineering, China University of Petroleum (East China), Qingdao 266580, PR China
[§]Institute for Advanced Study, Shenzhen University, Shenzhen 518060, PR China

**These authors contributed equally to this work.

*Qingshan Jason Niu: qjasonniu@szu.edu.cn; Armin Gölzhäuser: ag@uni-bielefeld.de.

S1. Defect fraction of CNM/PET

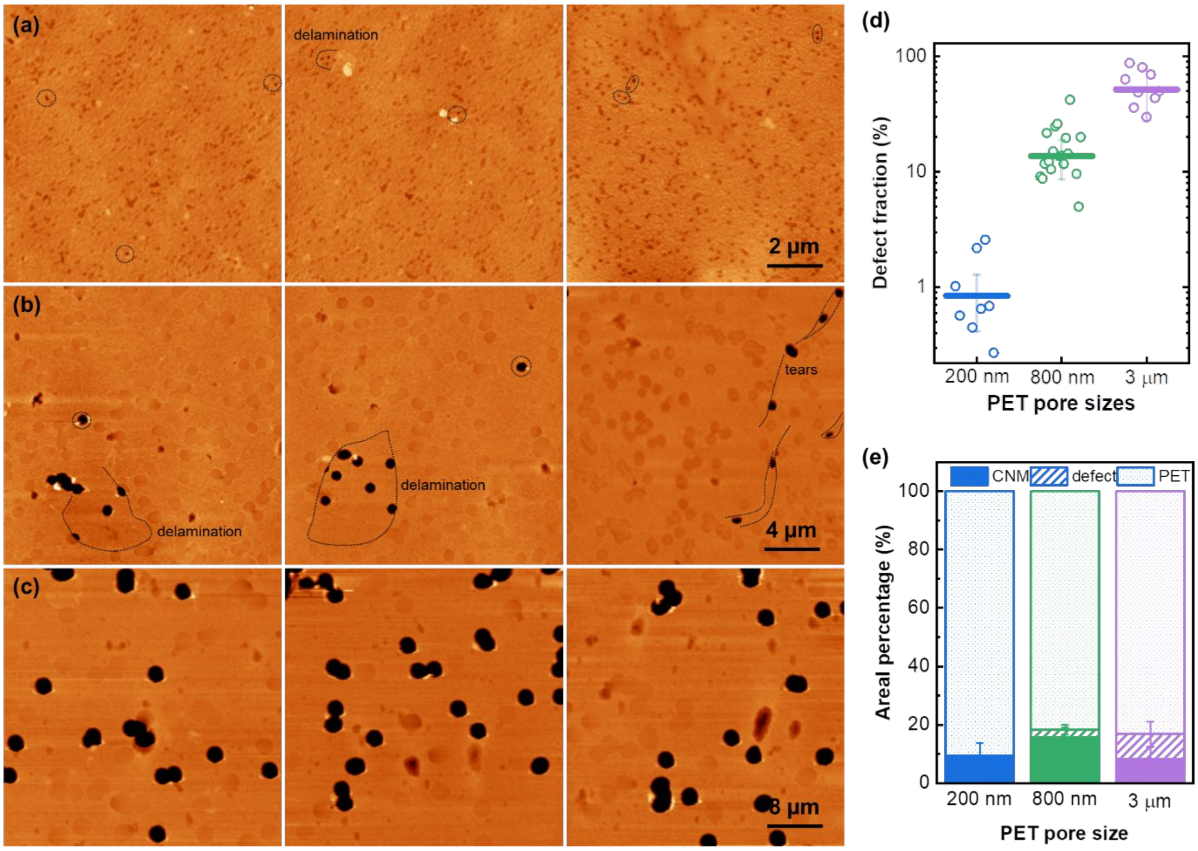

Figure S1. Representative AFM images of CNM/PET with PET pore sizes of (a) 200 nm, (b) 800 nm, and (c) 3 µm with some defects highlighted by dotted lines. (d) Defect fraction and (e) areal percentages of CNM, defects and PET for CNM/PET composites with different PET pore sizes as evaluated from AFM images.

S2. Forward osmosis measurements

S2.1 FO measurement with a membrane area of 0.2 cm^2

The 2 mL side-by-side glass cell used for the forward osmosis measurement with a membrane area of 0.2 cm^2 is schematically illustrated in **Figure 3(d)**. The composite membrane was sandwiched between the two glass cells with the CNM side facing the feed solution. Before each measurement, both feed and draw side cells were first filled with isopropanol followed by an exchange for DI water to avoid air bubbles trapped inside the glass orifice. Next, a 100 μL syringe was attached to one port of the feed cell and a Teflon plug was used to seal the other. To test the successful healing of the defects and to verify the leak-free connections in our system, we filled water to the uppermost level of the syringe, thereby creating a hydrostatic pressure of ~10 mbar (water column ≈ 0.1 m) across the membrane. For all the composites tested, we observed <3 μL water level drop in the syringe for a 30 min test, suggesting our system to be leak-free. Post the leak test, we exchanged the water in the draw side to NaCl solution of different concentrations for 20 min forward osmosis measurements. We tested 0.5 M NaCl solution first, followed by 0.25 M, 1 M and 0.75 M solutions. Between each measurements, the glass cell was thoroughly rinsed with DI water. Also, to ensure that no damage occurred during the measurements, we carried out additional leak test for each membrane before 1 M NaCl test and after 0.75 M NaCl test. **Figure S2** illustrates the recorded water level change as a function of time for a PA/CNM/PET composite tested with NaCl of different concentrations.

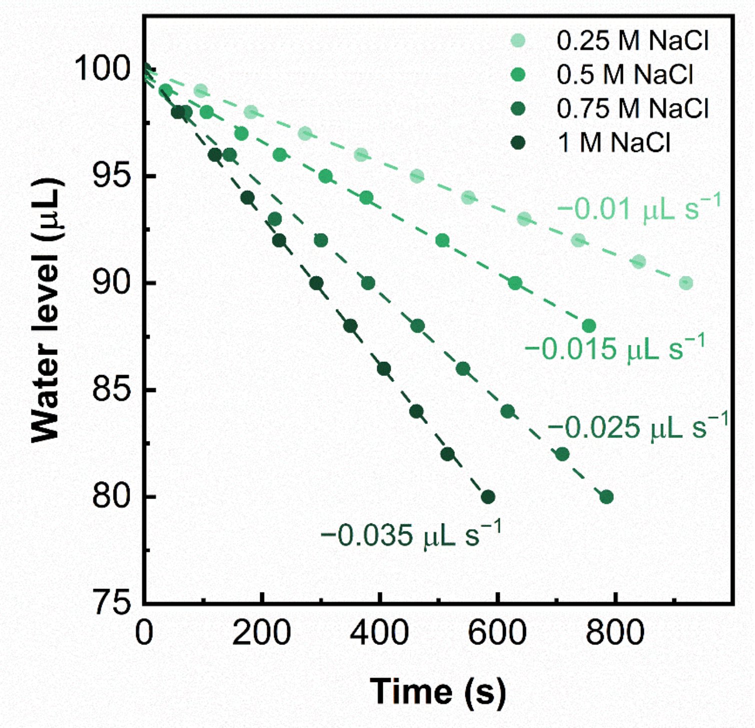

Figure S2. Water level drop as a function of time for a PA/CNMPET (800 nm pore) composite with 0.25 – 1 M NaCl as draw solution, *i.e.*, an osmotic pressure of 12 – 48 bar.

S2.2 Forward osmosis measurement with a membrane area of 3 cm^2

Figure S3(a, b) shows a schematic diagram of the cross-flow FO setup together with the home-built polycarbonate cell. Two peristaltic pumps were used to circulate the feed and draw solutions at a flow velocity of 0.1 m/s. Note that there was no transmembrane pressure and no spacers was applied. The water flux was determined at regular time intervals by measuring the weight loses of the FS with a digital mass balance and the reverse salt flux was quantified according to the FS concentration increase monitored by a conductivity sensor. Both the balance and the conductivity sensor are connected to a computer data logging system with a data acquisition rate of 0.05 s^{-1}. **Figure S3(c)** illustrates the water flux and reverse salt flux for PA/CNM/PET (800 nm pores) composites measured with the cross-flow setup as a function of time.

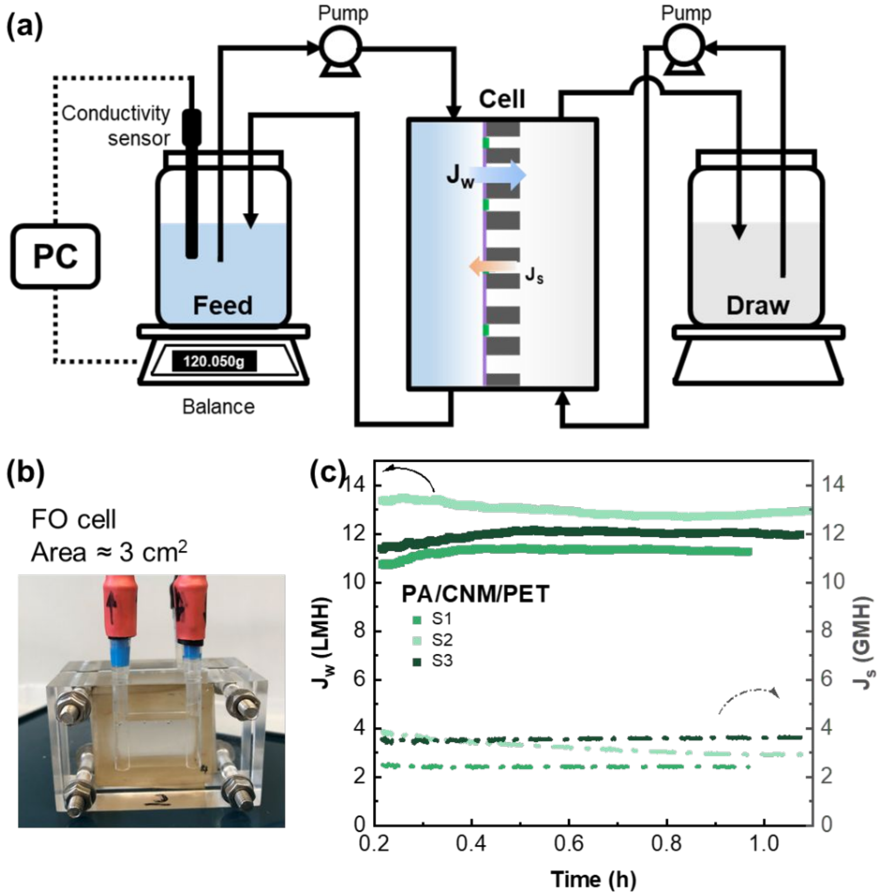

Figure S3. (a) Schematic diagram of the cross-flow forward osmosis set up and (b) the home-built polycarbonate cell with a membrane area of 3 cm^2. (c) Water and reverse salt fluxes for three PA/CNM/PET (800 nm pores) composites with 1 M NaCl as the draw solution and DI water as the feed solution.

S2.3 Influence of the membrane orientation

The orientation of the membrane, *i.e.*, CNM-facing-the-feed-solution (CNM-FS) or CNM-facing-the-draw-solution (CNM-DS), has an influence on the water flux and the reverse salt flux measured, as shown in **Figure S4**. In both glass cell and the cross-flow setup, we observed a higher water flux for the CNM-DS configuration, which is likely caused by the internal concentration polarization occurring inside the PET support.[1,2] When the draw solution is on the PET side, the dilution of the DS inside the support reduces the effective driving force, which then leads to a lower water flux.

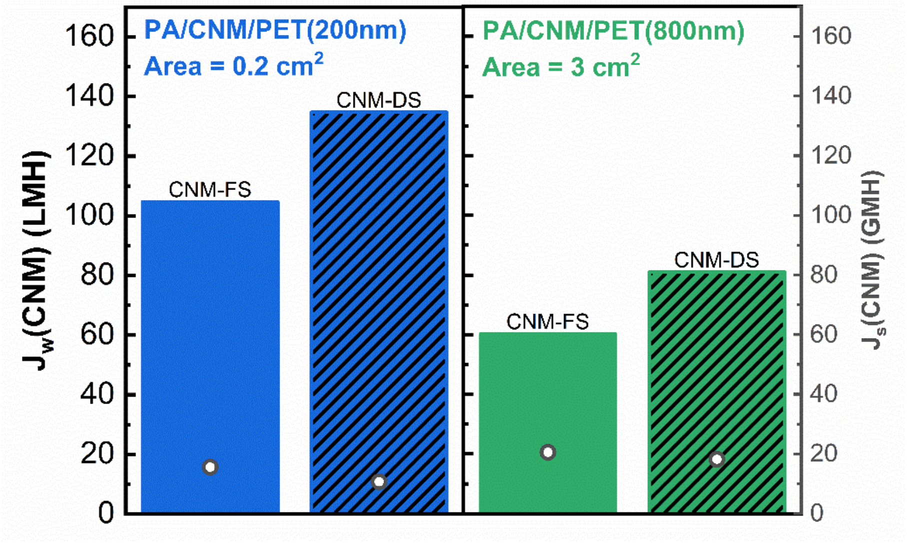

Figure S4. Influences of the membrane orientation, *i.e.*, CNM-facing-the-feed-solution (CNM-FS) or CNM-facing-the-draw-solution (CNM-DS) on the water flux and salt flux for PA/CNM/PET composites with different PET pore sizes and different measurement areas.

S2.4 Surface morphology post measurements

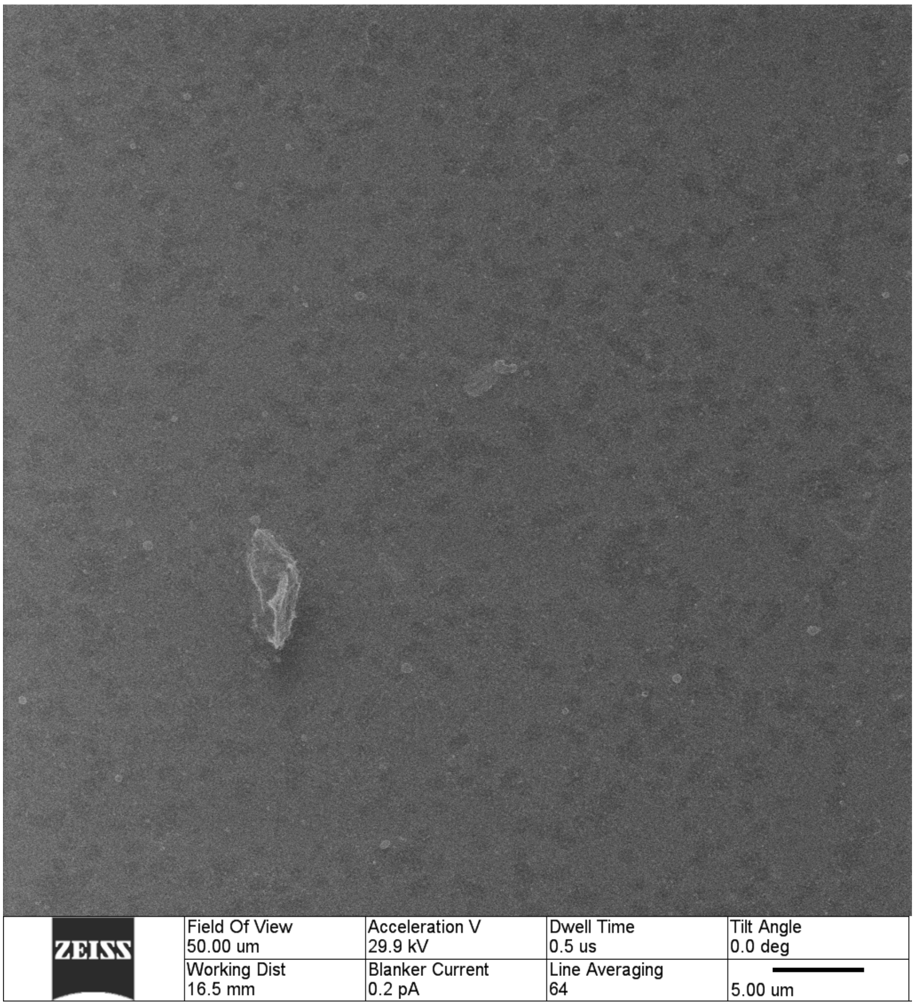

Figure S5. HIM image of PA/CNM/PET (800 nm pore) after FO measurements.

S3. Comparisons of PA/CNM/PET and PA/PET

Figure S6(a) compares morphologies of PA/PET composites with PET of different pore sizes. The ridge-and-valley or leaf-like structures, characteristic of PA,[3,4] slowly vanishes with the increase in PET pore size. This phenomenon is attributed to the decrease in the confinement provided by the support.[3] The corresponding water flux and reverse salt flux for PA/PET composites as well as the comparison to PA/CNM/PET composites are illustrated in **Figure S6(b)**. The water flux drops with the increasing PET pore size, which is likely due to the decrease in the roughness of the PA layer. It is noted that in all three cases, the PA/CNM/PET composite exhibits a higher water flux and a lower reverse salt flux, highlighting the superior performance of the CNM.

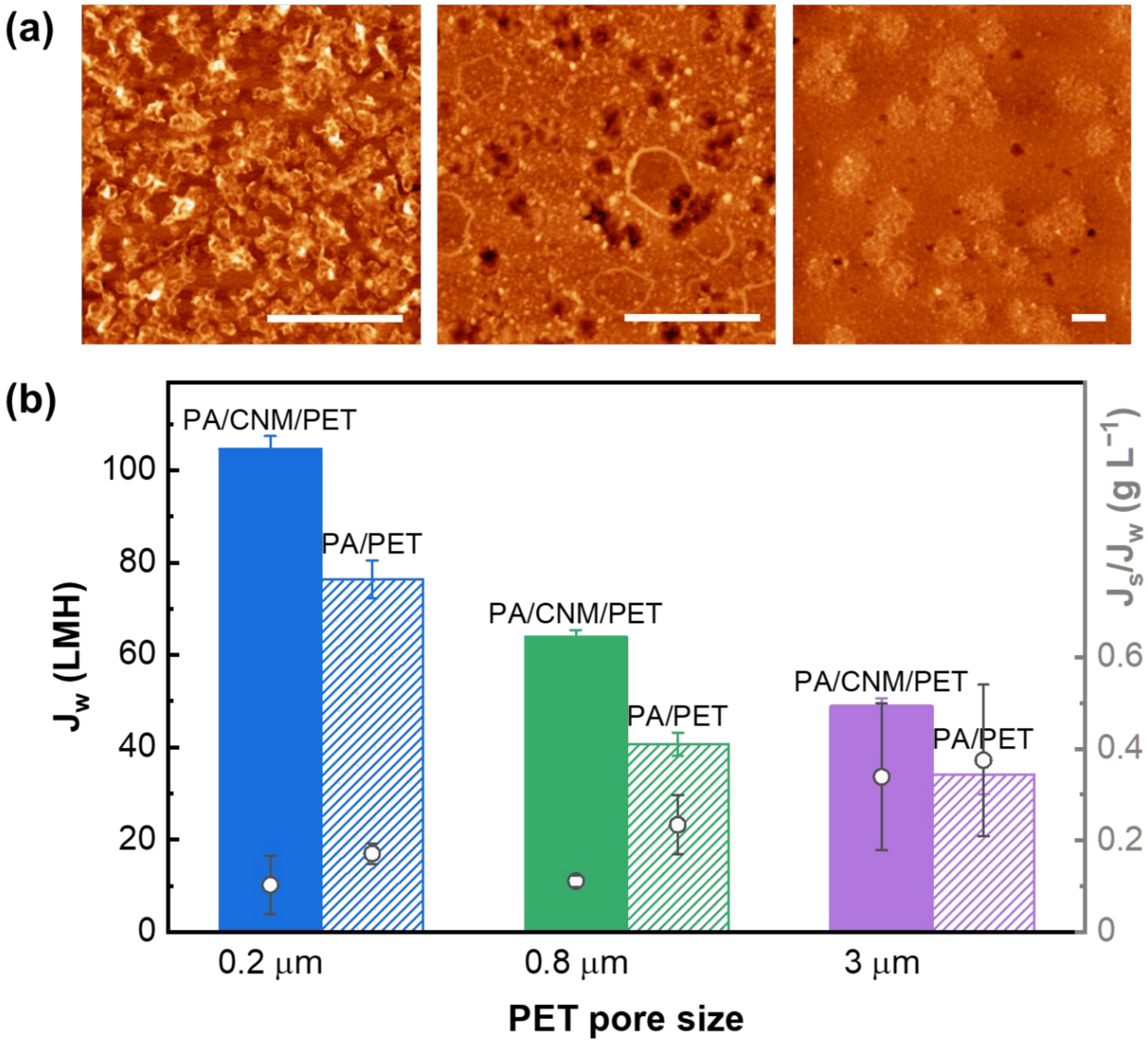

Figure S6. (a) AFM images of PA/PET composites with PET pore sizes of 0.2 μm, 0.8 μm and 3 μm, (scale bar = 4 μm). (b) Comparison of water flux (J_w) and specific reverse salt flux (J_s/J_w) for PA/CNM/PET and PA/PET composites with different PET pore sizes (membrane area ≈ 0.2 cm^2) The values are for freestanding membranes.

S4. Comparisons to other 2D materials based membranes

Table S1. Comparisons of water permeance and salt rejection of PA/CNM/PET composites with other 2D materials based FO membranes reported in the literature.

Membrane*	Area / cm^2	Support	Porosity of support / %	Active area / cm^2	Feed solution	Draw solution	Mode**	Volume / mL	Osmotic pressure / bar	Water permeance of active layer / LMH bar^{-1}	Water flux J_w / LMH	Water permeance P_w / LMH bar^{-1}	Salt flux J_s / GMH	J_s/J_w / g L^{-1}	Salt rejection / $(1\text{-}C_d/C_f)$%	Salt rejection / $(1\text{-}J_s/M/J_w/C_f)$%	Salt rejection (reverse osmosis) / %	Membrane thickness (nm)	References
PA/CNM/PET	0.2	PET (200nm pore)	9.4	0.019	DI H_2O	1M NaCl	AL-FS	2.5	48	2.18	9.94	0.207	1.024	0.103	-	-	-	1.2 + PA	This work
PA/CNM/PET	0.2	PET (800nm pore)	18.4	0.037	DI H_2O	1M NaCl	AL-FS	2.5	48	1.33	11.51	0.240	1.289	0.112	-	-	-	1.2 + PA	
PA/CNM/PET	0.2	PET (800nm pore)	18.4	0.037	20 mM NaCl	25 wt% Glycerol ethoxylate	AL-FS	2.5	17.2	0.71	2.26	0.131	-	-	-	99.8	-	1.2 + PA	
PA/CNM/PET	0.2	PET (3µm pore)	17.0	0.034	DI H_2O	1M NaCl	AL-FS	2.5	48	1.02	8.81	0.184	2.986	0.339	-	-	-	1.2 + PA	
PA/CNM/PET	3	PET (800nm pore)	18.4	0.552	DI H_2O	1M NaCl	AL-FS	2.5	48	1.37	12.12	0.253	2.930	0.242	-	-	-	1.2 + PA	
CNM 2×	0.05	PET (700nm pore)	5	0.0025	2 mM NaCl	1M NaCl	AL-FS	0.06	48	13.0	31.20	0.650	3.04	0.10	100	100	-	2.4	5
NG+IP (HMDA+APC)	0.24	PCTE (200nm pore)	10	0.024	16.6mM NaCl	26.47 wt% Glycerol ethoxylate	AL-FS	7.4 / 7.25	21.8	1.41	3.07	0.141	6.56	-	57.9	-120	-	0.34 + PA	6
NG+IP (HMDA+APC)	0.24	PCTE (200nm pore)	10	0.024	16.6mM $MgSO_4$	26.47 wt% Glycerol ethoxylate	AL-FS	7.4 / 7.25	22.0	1.41	3.10	0.141	0.89	-	94.3	70.4	-	0.34 + PA	
NG+IP (POSS+TMC)	0.20	PCTE (200nm pore)	9.4	0.018	16.6mM KCl	26.47 wt% Glycerol ethoxylate	AL-FS	8	20.3	3.41	6.50	0.321	0.64	-	97.2	89.9	-	0.34 + PA	7
NG+IP (POSS+TMC)	0.20	PCTE (200nm pore)	9.4	0.018	16.6mM NaCl	26.47 wt% Glycerol ethoxylate	AL-FS	8	20.3	3.41	6.50	0.321	0.57	-	97.5	91.0	-	0.34 + PA	
GNM/SWNT	0.16	PDMS on PET	19.6	0.031	DI H_2O	1M KCl	-	40	44.8	20.58	180.67	4.033	-	-	-	-	-	50	8
GNM/SWNT	0.16	PDMS on PET	19.6	0.031	DI H_2O	0.5M NaCl	-	40	25	22.00	107.80	4.312	-	-	-	-	-	50	
GNM/SWNT	0.16	PDMS on PET	19.6	0.031	0.5M KCl	2M sucrose	-	40	25	20.58	100.82	4.033	173.88	-	97.1	94.1	-	50	
GNM/SWNT	0.16	PDMS on PET	19.6	0.031	0.5M NaCl	2M sucrose	-	40	25	22.00	107.80	4.312	117.80	-	98.1	96.3	-	50	
GO-Gr	0.50	Anodisc alumina	-	-	0.1M NaCl	3M sucrose	-	25	75	-	0.53	0.007	3.02	5.66	97	3.25	-	5000	9
GO-Gr	0.50	Anodisc alumina	-	-	0.1M NaCl	3M sucrose	-	25	75	-	2.50	0.033	6.73	2.69	94	54	-	1000	
GO	0.50	Anodisc alumina	-	-	0.1M NaCl	3M sucrose	-	25	75	-	0.60	0.008	40.40	67.34	60	-1051	-	5000	
GO	0.05	Anodisc alumina	-	-	DI H_2O	1M sucrose	-	100	25	-	0.20	0.008	-	-	-	-	-	5000	10
GO	0.05	silver mesh (3µm pore)	-	-	DI H_2O	1M NaCl	-	-	48	-	0.11	0.002	126.36	1197	-	-	-	10000	11
GO/K^+	1.13	Al_2O_3	-	-	DI H_2O	0.25M NaCl	AL-DS	35	12	-	0.36	0.030	-	-	94.7	-	-	280	12
GO/K^+	1.13	Al_2O_3	-	-	DI H_2O	0.25M NaCl	AL-DS	35	12	-	0.23	0.019	-	-	96	-	-	550	

GO/K+	1.13	Al_2O_3	-	-	DI H_2O	0.25M NaCl	AL-DS	35	12	-	0.10	0.008	-	-	99	-	-	750	
GO	1.13	Al_2O_3	-	-	DI H_2O	0.25M NaCl	AL-DS	35	12	-	0.85	0.071	-	-	-	-	-	280	
GO	1.13	Al_2O_3	-	-	DI H_2O	0.25M NaCl	AL-DS	35	12	-	0.49	0.041	-	-	-	-	-	550	
GO	1.13	Al_2O_3	-	-	DI H_2O	0.25M NaCl	AL-DS	35	12	-	0.17	0.014	-	-	-	-	-	750	
rGO/K+	1.13	Al_2O_3	-	-	DI H_2O	0.25M NaCl	AL-DS	35	12	-	0.57	0.048	3.68	6.5	-	-	-	280	
rGO	1.13	Al_2O_3	-	-	DI H_2O	0.25M NaCl	AL-DS	35	12	-	0.22	0.018	17.61	80.0	-	-	-	280	
rGO	0.2	carbon tape	100	0.2	DI H_2O	0.5M NaCl	AL-FS	-	24	0.708	17	0.71	1.1	0.06	-	-	-	100	13
rGO	0.2	carbon tape	100	0.2	DI H_2O	1M NaCl	AL-FS	-	48	0.563	27	0.56	1.3	0.05	-	-	-	100	
rGO	0.2	carbon tape	100	0.2	DI H_2O	1.5M NaCl	AL-FS	-	72	0.556	40	0.56	1.3	0.03	-	-	-	100	
rGO	0.2	carbon tape	100	0.2	DI H_2O	2M NaCl	AL-FS	-	96	0.594	57	0.59	1.3	0.02	-	-	-	100	
rGO	0.2	carbon tape	100	0.2	0.1M NaCl	0.5M Ammonium bicarbonate	AL-FS	-	19.2	0.625	12	0.625	-	-		83.3	-	100	
Ethyl-2-ol-func. MoS_2	1	Nylon (220 nm pore)	-	-	0.1M NaCl	3M KCl	-	-	148	-	21.6	0.15	-	-	-	-	-	250	14
					0.1M NaCl	-	-	-	-	-	-	-	-	-	-	-	70.9		
Ethyl-2-ol-func. MoS_2	1	Nylon (220 nm pore)	-	-	0.1M NaCl	3M KCl	-	-	148	-	13.62	0.09	-	-	-	-	-	1000	
					0.1M NaCl	-	-	-	-	-	-	-	-	-	-	-	85.7		
acetamide-func. MoS_2	1	Nylon (220 nm pore)	-	-	0.1M NaCl	3M KCl	-	-	148	-	7.70	0.05	-	-	-	-	-	250	
					0.1M NaCl	-	-	-	-	-	-	-	-	-	-	-	77.9		
acetamide-func. MoS_2	1	Nylon (220 nm pore)	-	-	0.1M NaCl	3M KCl	-	-	148	-	5.33	0.04	-	-	-	-	-	1000	
					0.1M NaCl	-	-	-	-	-	-	-	-	-	-	-	85.7		
dye crystal violet (CV) decorated MoS_2	0.05	PVDF (100nm pores)	-	-	DI H_2O	1M sucrose	AL-DS	100	25	-	1	0.04	-	-	-	-	-	3000	15
dye crystal violet (CV) decorated MoS_2	0.05	PVDF (100nm pores)	-	-	DI H_2O	1M sucrose	AL-DS	100	25	-	0.3	0.012	-	-	-	-	-	6000	
dye crystal violet (CV) decorated MoS_2	0.05	PVDF (100nm pores)	-	-	1M NaCl	-	-	-	-	-	-	-	-	-	-	-	20	5000	
microscale pG	2×10^{-7}	SiRN	100	2×10^{-7}	DI H_2O	1M KCl	-	48	48	262.5	12600	252	-	-	100	100	-	0.34	16
microscale CNM	6.4×10^{-7}	SiRN	100	6.4×10^{-7}	DI H_2O	-	-	-	-	750	-	750	-	-	-	-	-	1.2	17

*NG+IP refers to nanoporous graphene (NG) with defects sealed by interfacial polymerization (IP) of hexamethylene diamine (HMDA) + adipoyl chloride (APC) or octa-ammonium polyhedral-oligomeric-silsesquioxane (POSS) + trimesoyl chloride (TMC); GNM/SWNT refers to graphene nanomesh (GNM) supported by an interwoven network of single-walled carbon nanotubes (SWNTs); GO-Gr refers to graphene oxide-graphene membranes; rGO refers to reduced graphene oxide; (r)GO/K+ refers to (reduced) graphene oxide membranes with K^+ ion controlling the interlayer spacing.

**AL-FS = active-layer-facing-the-feed-solution; AL-DS = active-layer-facing-the-draw-solution.

References

(1) Wei, J.; Qiu, C.; Tang, C. Y.; Wang, R.; Fane, A. G. Synthesis and Characterization of Flat-Sheet Thin Film Composite Forward Osmosis Membranes. *J. Memb. Sci.* **2011**, *372* (1–2), 292–302. https://doi.org/10.1016/j.memsci.2011.02.013.

(2) Tang, C. Y.; She, Q.; Lay, W. C. L.; Wang, R.; Fane, A. G. Coupled Effects of Internal Concentration Polarization and Fouling on Flux Behavior of Forward Osmosis Membranes during Humic Acid Filtration. *J. Memb. Sci.* **2010**, *354* (1–2), 123–133. https://doi.org/10.1016/j.memsci.2010.02.059.

(3) Peng, L. E.; Yao, Z.; Yang, Z.; Guo, H.; Tang, C. Y. Dissecting the Role of Substrate on the Morphology and Separation Properties of Thin Film Composite Polyamide Membranes: Seeing Is Believing. *Environ. Sci. Technol.* **2020**, *54* (11), 6978–6986. https://doi.org/10.1021/acs.est.0c01427.

(4) Peng, L. E.; Yang, Z.; Long, L.; Zhou, S.; Guo, H.; Tang, C. Y. A Critical Review on Porous Substrates of TFC Polyamide Membranes: Mechanisms, Membrane Performances, and Future Perspectives. *J. Memb. Sci.* **2022**, *641* (September 2021), 119871. https://doi.org/10.1016/j.memsci.2021.119871.

(5) Yang, Y.; Hillmann, R.; Qi, Y.; Korzetz, R.; Biere, N.; Emmrich, D.; Westphal, M.; Büker, B.; Hütten, A.; Beyer, A.; Anselmetti, D.; Gölzhäuser, A. Ultrahigh Ionic Exclusion through Carbon Nanomembranes. *Adv. Mater.* **2020**, *32* (8), 1907850. https://doi.org/10.1002/adma.201907850.

(6) O'Hern, S. C.; Jang, D.; Bose, S.; Idrobo, J. C.; Song, Y.; Laoui, T.; Kong, J.; Karnik, R. Nanofiltration across Defect-Sealed Nanoporous Monolayer Graphene. *Nano Lett.* **2015**, *15* (5), 3254–3260. https://doi.org/10.1021/acs.nanolett.5b00456.

(7) Cheng, P.; Kelly, M. M.; Moehring, N. K.; Ko, W.; Li, A. P.; Idrobo, J. C.; Boutilier, M. S. H.; Kidambi, P. R. Facile Size-Selective Defect Sealing in Large-Area Atomically Thin Graphene Membranes for Sub-Nanometer Scale Separations. *Nano Lett.* **2020**, *20* (8), 5951–5959. https://doi.org/10.1021/acs.nanolett.0c01934.

(8) Yang, Y.; Yang, X.; Liang, L.; Gao, Y.; Cheng, H.; Li, X.; Zou, M.; Ma, R.; Yuan, Q.; Duan, X. Large-Area Graphene-Nanomesh/Carbon-Nanotube Hybrid Membranes for Ionic and Molecular Nanofiltration. *Science (80-.).* **2019**, *364* (6445), 1057–1062. https://doi.org/10.1126/science.aau5321.

(9) Abraham, J.; Vasu, K. S.; Williams, C. D.; Gopinadhan, K.; Su, Y.; Cherian, C. T.; Dix, J.; Prestat, E.; Haigh, S. J.; Grigorieva, I. V.; Carbone, P.; Geim, A. K.; Nair, R. R. Tunable Sieving of Ions Using Graphene Oxide Membranes. *Nat. Nanotechnol.* **2017**, *12* (6), 546–550. https://doi.org/10.1038/nnano.2017.21.

(10) Joshi, R. K.; Carbone, P.; Wang, F. C.; Kravets, V. G.; Su, Y.; Grigorieva, I. V.; Wu, H. A.; Geim, A. K.; Nair, R. R. Precise and Ultrafast Molecular Sieving through Graphene Oxide Membranes. *Science (80-.).* **2014**, *343* (6172), 752–754. https://doi.org/10.1126/science.1245711.

(11) Deng, M.; Kwac, K.; Li, M.; Jung, Y.; Park, H. G. Stability, Molecular Sieving, and Ion Diffusion Selectivity of a Lamellar Membrane from Two-Dimensional Molybdenum Disulfide. *Nano Lett.* **2017**, *17* (4), 2342–2348. https://doi.org/10.1021/acs.nanolett.6b05238.

(12) Chen, L.; Shi, G.; Shen, J.; Peng, B.; Zhang, B.; Wang, Y.; Bian, F.; Wang, J.; Li, D.; Qian, Z.; Xu, G.; Liu, G.; Zeng, J.; Zhang, L.; Yang, Y.; Zhou, G.; Wu, M.; Jin, W.; Li, J.; Fang, H. Ion Sieving in Graphene Oxide Membranes via Cationic Control of Interlayer Spacing. *Nature* **2017**, *550* (7676), 380–383. https://doi.org/10.1038/nature24044.

(13) Liu, H.; Wang, H.; Zhang, X. Facile Fabrication of Freestanding Ultrathin Reduced Graphene Oxide Membranes for Water Purification. *Adv. Mater.* **2015**, *27* (2), 249–254. https://doi.org/10.1002/adma.201404054.

(14) Ries, L.; Petit, E.; Michel, T.; Diogo, C. C.; Gervais, C.; Salameh, C.; Bechelany, M.; Balme, S.; Miele, P.; Onofrio, N.; Voiry, D. Enhanced Sieving from Exfoliated MoS2 Membranes via Covalent Functionalization. *Nat. Mater.* **2019**, *18* (10), 1112–1117. https://doi.org/10.1038/s41563-019-0464-7.

(15) Hirunpinyopas, W.; Prestat, E.; Worrall, S. D.; Haigh, S. J.; Dryfe, R. A. W.; Bissett, M. A. Desalination and Nanofiltration through Functionalized Laminar MoS2 Membranes. *ACS Nano* **2017**, *11* (11), 11082–11090. https://doi.org/10.1021/acsnano.7b05124.

(16) Surwade, S. P.; Smirnov, S. N.; Vlassiouk, I. V; Unocic, R. R.; Veith, G. M.; Dai, S.; Mahurin, S. M. Water Desalination Using Nanoporous Single-Layer Graphene. *Nat. Nanotechnol.* **2015**, *10* (5), 459–464. https://doi.org/10.1038/nnano.2015.37.

(17) Yang, Y.; Dementyev, P.; Biere, N.; Emmrich, D.; Stohmann, P.; Korzetz, R.; Zhang, X.; Beyer, A.; Koch, S.; Anselmetti, D.; Gölzhäuser, A. Rapid Water Permeation Through Carbon Nanomembranes with Sub-Nanometer Channels. *ACS Nano* **2018**, *12* (5), 4695–4701. https://doi.org/10.1021/acsnano.8b01266.
